# Quacks Universally Advertise

**Published:** 1851-10

**Authors:** 


*Quacks Universally Advertise.*—We do not mean by this, that all who advertise are bearers of the above title, because many skillful and talented men very properly insert their cards for the benefit of strangers seeking their assistance, but men who seek to draw business by their impudent bombast and falsehood, are in the last stage of charlatanism. Nor would we pretend to say that it is a sign of a man's genuine talent who is free from this professional crime. There is hardly a paper in the land which does not contain some astonishing announcement of the insertion or completion of some unequalled performance, either in operative surgery or mechanical dentistry. Now, it is such physicians and dentists that we denounce as quacks, sharpers and grinders. If they were honest men, and really possessed the pretended skill, they need no such artifice. Were such operations performed, have we not enough medical and surgical journals, the only proper medium for such announcements, which are always open for the introduction of every evidence of talent, and where the operator would receive the first, best acknowledgment of his ability. But a quack and his dollar will paste himself on every corner of the streets. The more refined rogue will avail himself of the popular papers of the day, or some ladies' repository, to announce wonders never performed, and abilities never possessed. The still more subtle *nondescript*, refines his duplicity under the garb of mock praise, and condemns the slandering qualities of some venerable dame, he never heard of, who has denounced Dr. So-and-So, and who has determined to abandon the "old school for the more modern and approved homeopathy, hydropathy, or some other undefinable absurdity. What can be more offensive to the man of a refined professional education, and what more distinctive mark can be required to designate men with and without abilities. With this, we introduce two specimens recently received, and give them to our readers for a moment's amusement, but with the sincere hope that such monstrous licenses will be crushed, and that suffering humanity will at last safely rely upon the honesty of its proffered relief. We give the following article entire, from the Boston Medical and Surgical Journal, perfectly agreeing, as we do, with the editorial expression of such things.

*How Quacks Advertise.*—Below we give part of the advertisement of a notorious individual, as a specimen of the way of doing business among practitioners of his class. It is quite time their trickery was exposed to the public. We are aware that it is the opinion of many in our ranks, that it is better to let such matters alone; but we must differ from them in regard to the expediency of such a course. A written answer to the following questions is to form the basis of a diagnosis of any AIL a patient may have.

"Give name, age, residence, occupation; family consumptive, or what complaints subject to; where born and brought up; married or single; strong or delicate; lean or fleshy; tall or short, straight or stooping, or deformed; height and size around the waist two inches above the hips; color of hair; complexion; have you any humor, scrofula, cancer, skin disease, head-ache, cough, asthma, rheumatism, or pain any where, loss of voice, hoarseness, catarrh, dropsy, expectorate much, raise blood, fever or night sweats, chills, confined to bed or house, palpitation, nervous, fits, palsy, bad dreams, sour or sick stomach, dyspepsia, flatulence, distress at stomach, colic, all-gone feeling any where, costive, diarrhea, appetite good or bad, piles, fistula, gravel, heat of urine or scanty or sediment? If a lady—married? had any children? any female complaints? irregularity? weak back? pain any where? any bloating? dropsy? bilious? worms? indigent or easy circumstances? any bad fits of sickness? doctor much?

"As it is a source of pleasure to Dr. ——— to alleviate the sufferings of the invalid, he has concluded in future to make no charge for OFFICE consultation and examination of the chest."

The inquiry as to the pecuniary resources of a patient is a very important one, and we wonder the wily doctor should have placed it so near the bottom of the list. It is understood that these questions are propounded to those who may live at a distance, and cannot conveniently visit the great Æsculapius in propria personæ.

